# ArcMAP – ML assisted medical concept mapping to accelerate NHS data standardization

**DOI:** 10.3389/fdgth.2026.1770903

**Published:** 2026-04-14

**Authors:** Joseph Cronin, Olivia Wiper, Anthony Poncet, Keiran Tait, Bernard Cooke, Andrew Fry, Janie Baxter, Robert Dürichen

**Affiliations:** Arcturis Data, Kidlington, United Kingdom

**Keywords:** data standardisation, electronic health records, natural language processing, OMOP, real world data

## Abstract

The increasing use of electronic health records (EHRs) for real-world evidence (RWE) studies is hindered by substantial heterogeneity in data collection practices and local coding schemes across healthcare providers. Data standardization—particularly the mapping of locally defined medical concepts to standardized vocabularies—is therefore a critical but labour-intensive step, traditionally relying on extensive manual review by clinical experts. While a range of machine-learning (ML) approaches have been proposed to support medical concept mapping, their integration into practical, end-to-end workflows and their performance under real-world conditions remain insufficiently studied. In this work, we present ArcMAP, an end-to-end application that integrates a state-of-the-art biomedical representation model (BioLORD) into a human-in-the-loop workflow designed to streamline and accelerate medical concept mapping. ArcMAP provides a graphical user interface that enables clinical experts to efficiently review, validate, and correct automated mapping suggestions. A core component of the system is a continuous learning pipeline, in which expert feedback is systematically captured and used to update the underlying model, allowing ArcMAP to adapt to evolving coding practices and newly onboarded data sources. We conduct a comprehensive evaluation of ArcMAP across multiple deployment scenarios, including the impact of continuous fine-tuning, the onboarding of a new hospital, and a longitudinal real-world evaluation conducted over a two-month period using medication and laboratory test data from five UK-based NHS hospitals. Our results demonstrate the importance of domain-specific fine-tuning, with top-1 accuracy for laboratory test names increasing from 37.0% to 91.6%. However, when simulating the onboarding of a new hospital, the system achieves a weighted average top-1 accuracy of only 73.5%, indicating substantial variability across NHS hospital systems. In real-world use, the use of ArcMAP indicates an increased mapping efficiency compared to manual workflows, while also revealing considerable variation across individual data-mapping sessions.

## 1 Introduction

Electronic health records (EHRs) are an extensive and increasingly important source of data for generating real-world evidence (RWE). Common applications of such studies include retrospective cohort analyses, comparison of treatment effectiveness, and the development of external control arms (1). Utilizing real world healthcare data can, however, prove challenging when used for research purposes. This is due to the inherent and uncontrolled biases and confounding factors associated with data gathered in a hospital system. Additionally, there are large variations in the way that data is collected and managed between healthcare providers, as each source may have their own electronic patient record system that records and encodes data from routine medical encounters in a unique way. Without the ability to pool together large amounts of data from disparate sources using data standardization, real world data cannot be used for large-scale RWE studies (2).

**Figure 1:**
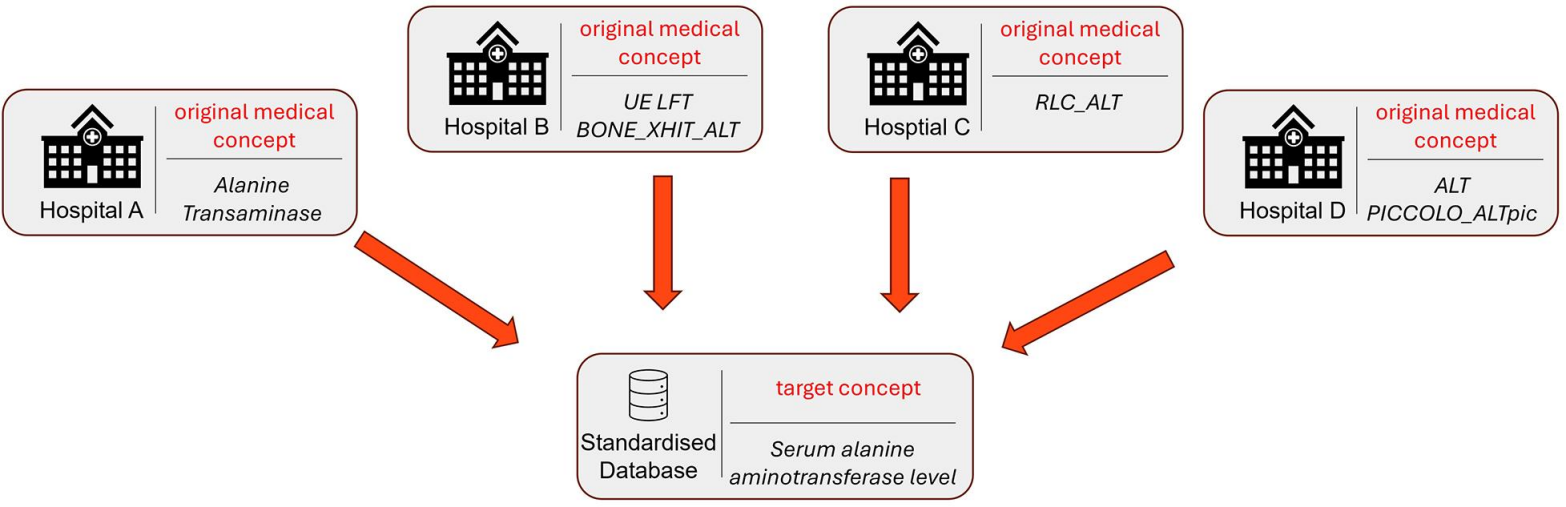
Illustrative example of the data standardization problem. Different hospital systems provide 4 different versions of the medical concept “*serum alanine aminotransferase level”.*

The data standardization process consists of two main parts: mapping the data schema of the source data to a common data model, and mapping each of the individual medical concepts (e.g., names of specific laboratory tests or medications) to a common medical concept. This latter step is crucial to ensure high-quality RWE studies but is also cumbersome, as it traditionally requires clinical experts to manually review each medical concept and map it to a common concept (3). An example of this can be seen in Figure 1, which shows four different medical concepts received from different NHS hospitals, which all refer to the same standardized medical concept “Serum alanine aminotransferase level”. As illustrated, some of the original medical concepts are very close to the target concept, e.g., “Alanine Transaminase”, however others require an in-depth analysis combined with expert knowledge to correctly infer the right standardized concept. One such tool introduced by the Observational Health Data Sciences and Informatics (OHDSI) community is USAGI, an open-source software application that automates vocabulary mapping by using term frequency–inverse document frequency (TF-IDF) similarity computations [“documentation:software:usagi (Observational Health Data Sciences and Informatics),” n.d.].

Selecting an appropriate target vocabulary adds another layer of complexity, as the healthcare domain offers hundreds of options with varying levels of granularity (4). The correct target vocabulary should be always defined by the research objectives. For example, SNOMED CT (Systematized Nomenclature of Medicine – Clinical Terms) is a broadly accepted ontology but contains over one million concepts, many of which are very specific or might be irrelevant for the specific use case. Conversely, many vocabularies are not sufficiently granular, leading to potential data loss when mapping source data to common concepts. Additional problems relating to target vocabularies include lack of ontology coverage for the breadth of concepts within the dataset and context dependence for polysemous terms (5, 6). A widely utilized clinical data model is the OMOP CDM (Observational Medical Outcomes Partnership Common Data Model), which has been adopted for a growing number of RWE healthcare studies (7, 8). The OMOP CDM uses standardized vocabularies for medical concepts such as LOINC (Logical Observation Identifiers Names and Codes) and SNOMED CT, allowing researchers to be flexible in selecting which target ontologies to map data against.

The use of NLP based methods has been widely explored in relation to standardizing free-text clinical data (9, 10) and mapping medical concepts to structured ontologies (11, 12) in research environments, however there has been little work on integrating these methods into a large-scale continuous learning pipeline. Techniques in the field range from simple rule-based systems to more sophisticated machine learning methods. Rule-based systems offer high precision provided that the rules are well defined, however the generation of appropriate rules can be time consuming, and they do not generalize well to new data sources. Kang et al. introduced an automated terminology mapping pipeline based on a deep learning approach, reporting at least a 10% improvement in matching accuracy compared to Usagi (13). Since the development of transformer-based neural networks in 2019, most notably BERT (25), a wide range of NLP applications have emerged in the healthcare domain. These models, often referred to as small language models (SLMs), have been widely adopted for tasks such as medical concept representation and data standardization. Within this context, several BERT-based models have been proposed that differ primarily in how biomedical knowledge is incorporated. SapBERT focuses on aligning synonymous concept names using metric learning over ontology-derived synonym sets, enabling nearest-neighbour-based normalization (14). CODER extends this approach by additionally leveraging relational information from biomedical knowledge graphs to shape the embedding space beyond synonymy alone (15). In contrast, KRISSBERT adopts a contextualized formulation of entity linking, learning representations of mentions in context via self-supervised contrastive learning on large unlabeled corpora, thereby addressing ambiguity in real-world text (16). BioLORD-2023 follows a complementary strategy by grounding concept representations in textual definitions and ontology-derived descriptions, combining contrastive learning with distillation to support robust biomedical concept representations (17).

Recently, the emergence of large language models (LLMs) has driven increased interest in generative approaches to medical data standardization and concept mapping (18–20). These methods demonstrate strong zero-shot and few-shot capabilities and can operate effectively without requiring large task-specific training datasets. However, their generative nature also makes LLM-based systems susceptible to hallucinations, potentially producing fluent but incorrect mappings when domain knowledge is incomplete or ambiguous (21). To mitigate this limitation, recent work by (18) adopts a retrieval-augmented generation (RAG) framework, grounding LLM outputs in external controlled vocabularies and structured knowledge sources to improve reliability and reduce spurious predictions. Despite these advances, deploying LLM-based solutions typically requires access to high-performance GPU resources or cloud-based infrastructure, which can limit their practicality and increase operational costs in many healthcare and research environments.

In this paper, we present ArcMAP (Arcturis MAPping), a novel machine learning-based data standardization tool. ArcMAP uses a SLM prediction algorithm and has the capability to transform source medical concepts that are specific to a given health provider, such as an NHS trust or specific hospital, into the most likely common medical concepts, such as SNOMED CT or others. This simplifies the task for a clinical expert who needs only to review rather than manually map each concept and intervene if the prediction is incorrect. This makes the process more efficient compared to the manual approach. The prediction model in ArcMAP has the capability to continuously learn with each new reviewed mapping, enabling the tool to improve over time (18). This allows data from new sources to be added more easily, enabling better inference of the correct standardized concept based on its knowledge of previous writing and coding styles.

Following a brief overview of ArcMAP and its core functionality, the main contributions of this paper are:

- **Model evaluation:** A comprehensive assessment of the ArcMAP prediction model and various other baseline small and large machine learning models for the task of data standardization; evaluating different scenarios like continuous learning and onboarding of a new hospital.
- **Real-world evaluation:** Detailed insights and lessons learned from deploying ArcMAP in a production environment, from both technical and health informatics perspectives.

## 2 Materials and methods

### 2.1 Overview of ArcMAP

ArcMAP is a tool designed to support and streamline the mapping of source concept codes to target concepts by leveraging the expertise of clinically trained professionals, such as health informaticians. The system comprises three core components: a graphical user interface (GUI), a prediction algorithm, and an underlying database.

**Figure 2:**
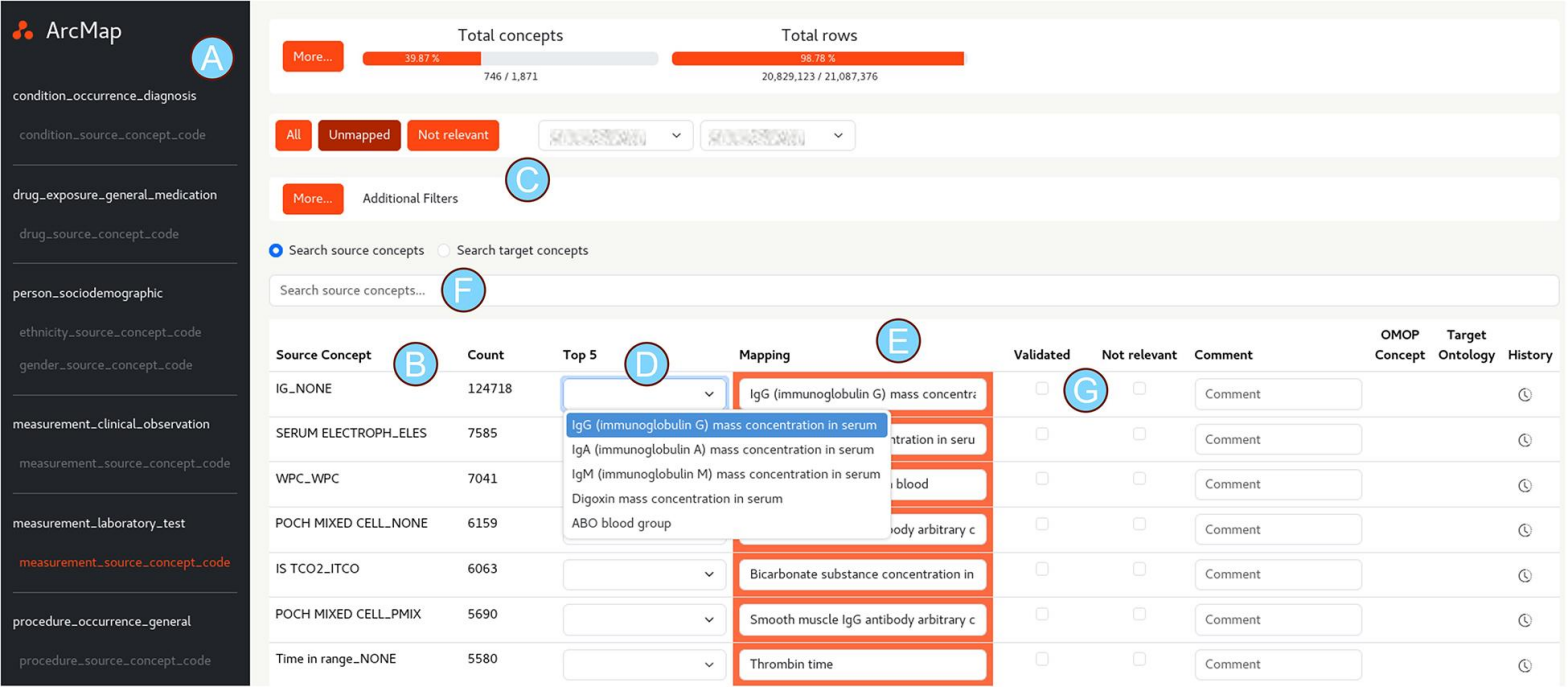
Graphical user interface of the ArcMAP app and its core functionality. A: selection of clinical data modality; B: Source concept and frequency; C: Filter options e.g., display only source concepts without a mapping; D: source concept specific prediction of the closest target concepts; E: selected target concept; F: search functionality to find specific source or standardized concepts; G: Option to validate or marks concept as “not relevant”.

The GUI is shown in Figure 2 and marker A-G illustrate the functionality of the application. A: The users begin by selecting a specific medical data type (e.g., laboratory tests or medication names). All source concepts available in the database are then displayed, sorted by frequency (B), with optional filters to e.g., exclude concepts that have already been mapped (C). For each source concept, a drop-down menu presents the top five closest matches generated by the ArcMAP prediction algorithm (D), alongside an input field for entering the appropriate target concept manually if required (E). The algorithm's best match is preselected by default. If the desired target concept is not among the suggested options, users can perform a manual search using string matching by overwriting the suggestions (F).

**Figure 3:**
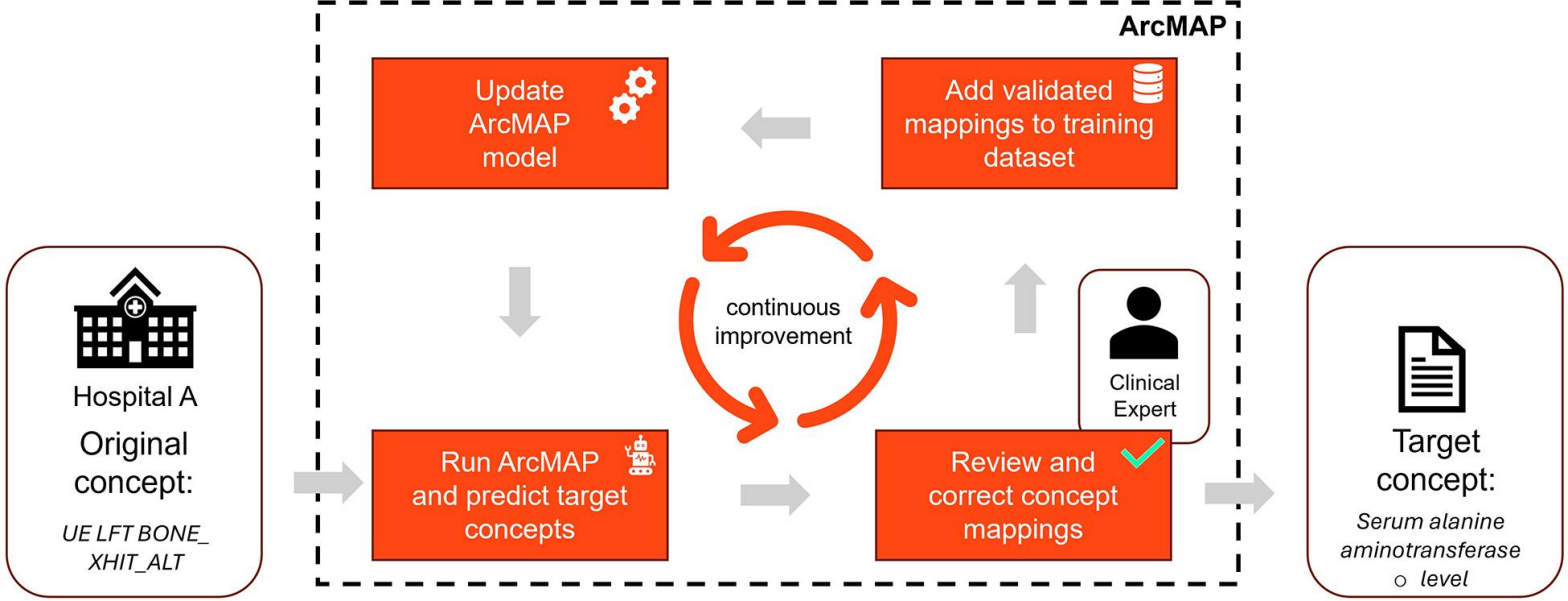
Schematic of continuous learning pipeline. Raw concept names are ingested, and the most likely target concepts are predicted using the ArcMAP model. These predictions are reviewed and validated by a clinical expert. Reviewed mappings then serve as additional training data for the model to help improve accuracy.

Once a suitable mapping is identified, the user can validate the selection (G). Alternatively, the concept may be marked as *not relevant*, indicating that it does not require mapping based on the user's judgement. At the end of each mapping session, all validated mappings are exported to the database and integrated into the data standardisation pipeline, which transforms and harmonises source data from various hospital systems (Figure 3). The updated model is applied automatically in future sessions, ensuring that ArcMAP evolves and learns with each mapping iteration.

### 2.2 ArcMAP prediction model

The objective of the ArcMAP prediction model is to accurately map a source concept to its corresponding target concept from a set of chosen ontologies. This is achieved by computing the cosine similarity between source and target concept embeddings. Concept embeddings were generated directly from raw source and target concept names, without enriching the concepts with further description. Following the work from (18), this would be categorised as an atomic mapping, i.e., the mapping of single entities without considering further attributes (e.g., hierarchy). Preprocessing was applied to raw concept names by converting to lower case, removing special characters (excluding “%”) and trailing whitespace. For example, a raw source concept string of “Platelets_B_%” would be input to the model as “platelets b %”.

The ArcMAP prediction model builds upon BioLORD-2023 (referred to as BioLORD), a state-of-the-art biomedical language representation model. This BERT based transformer model employs a novel pre-training strategy designed to generate semantically rich embeddings for clinical sentences and biomedical concepts, and has demonstrated superior performance in concept similarity and entity linking tasks (17). For ArcMAP, BioLORD was further finetuned using source-target concept mapping pairs to optimise performance for local data.

Finetuning was performed using a contrastive loss function, where training samples consisted of both positive and negative source–target concept pairs. For each source concept, a set of *n_pos_* positive examples (correct mappings) and *n_neg_* negative examples (incorrect mappings) was selected. Incorrect mappings were sampled randomly from the set of all target concepts excluding the correct mapping. During training, embeddings of source concepts were adjusted to move closer to positive examples and further away from negative ones.

While training the model requires the use of a GPU, generating concept embeddings can be run easily on a regular CPU machine. Importantly, the model is run over all available source and target concepts in one pass, and generated embeddings are stored in a backend database. This means that at inference time, the ArcMAP app only needs to compute the cosine similarity between embeddings in order to predict the most likely target concepts for a given source concept. Concepts with higher similarity scores are considered better matches. For the ArcMAP use case, cosine similarities are computed across all source–target concept pairs within the selected data type, and the top five closest common concepts are presented to the user as mapping suggestions.

### 2.3 Dataset and target vocabularies

This study used anonymised EHR data from the Arcturis Real-World Data Network research database, which has received ethical approval from the NHS Health Research Authority Yorkshire & The Humber – Leeds East Research Ethics Committee (REC) (reference: 24/YH/0164). EHR data was obtained from five NHS hospitals based in the United Kingdom. The dataset contains a total of 14,200 source medication names and 53,084 source laboratory test names. Of these source concepts, 6,179 medication names and 2,261 laboratory test concepts were mapped to a single target concept from a corresponding ontology through a manual process by health informaticians without the use of ArcMAP. 85.3% of medication names and 99.8% of laboratory tests were unique to a single hospital, with the remaining percentage of concepts appearing across multiple trusts. Medication names were mapped against the Virtual Therapeutic Moiety (VTM) ontology, part of the dm+d (dictionary of medicines and devices) system, which categorizes medications at varying levels of granularity. VTM is the most generic classification, while more specific categories exist, such as AMPP. This system aids in standardizing clinical concepts across healthcare datasets, facilitating accurate mapping of medications to their corresponding codes in systems like SNOMED CT. Laboratory test names were mapped against the Pathology and Laboratory Medicine (PaLM) observable entity simple reference set, a subset of SNOMED CT codes used as the standard for coding laboratory tests within the NHS. PaLM has replaced the outdated Pathology Bounded Code List (PBCL) and provides a more granular approach to laboratory test coding, facilitating better data management and interoperability in healthcare settings.

### 2.4 Experimental setup

#### 2.4.1 Experiment 1 - model evaluation

The objective of this experiment was to evaluate the performance of the ArcMAP prediction algorithm against several baseline methods for the task of data standardisation under different scenarios.

**Comparison Models:** ArcMAP was compared with three domain-specific models standard in concept normalization, SapBERT (14), KRISSBERT (16) and CODER (15), as well as two larger, general-purpose embedding models, Qwen3-0.6B (27) and EmbeddingGemma-300M (26), selected based on their ranking on the Massive Text Embedding Benchmark leaderboard (24). To ensure comparability, embedding models with more than 1 billion parameters were excluded from this analysis.

In addition, given the great success of LLMs in recent years, a generative model was evaluated as an alternative approach to the embeddings-based data standardization pipeline. Here, a Claude 3.7 Sonnet model was supplied with a complete list of target concepts and prompted to return the 5 most likely target concepts for a given source concept. Full prompt details are provided in Supplementary Figure 2. As the context window of the Claude 3.7 sonnet model is 200,000 tokens, the entire target concept could be added to the context simultaneously without the need of batching or RAG approaches. This model was accessed via AWS Bedrock and was run with *temperature*=0.01 and *top_p_*=1 parameters to enforce output to be as deterministic as possible. While the model introduces significant costs and additional hardware constraints, it is important to investigate if there is a benefit to using a current-state-of-art LLM.

**Evaluation Metrics:** All models were evaluated in the different scenarios with respect to these metrics:

- Top-1 Accuracy (*acc_top1_*)**:** The highest-ranked predicted target concept matches the correct concept in the ground truth.
- Top-5 Accuracy (*acc_top5_*)**:** The correct target concept appears within the top five predicted concepts.
- Top-1 Accuracy (frequency weighted)**:** Top-1 accuracy weighted by how frequently a given concept appears in the data
- Top-5 Accuracy (frequency weighted)**:** Top-5 accuracy weighted by how frequently a given concept appears in the data
- Mean reciprocal rank (MRR): The average reciprocal ranks of all predicted concepts
- Normalised Discounted Cumulative Gain (NDCG@5): A ranking quality metric that compares rankings to an ideal order where all relevant items are at the top of the list

**Scenarios:**

*Scenario 1 (S1) – Baseline Performance:* To assess baseline performance, embedding models were evaluated on all available medication names and laboratory test names without any finetuning.

*Scenario 2 (S2) – Iterative Fine-Tuning:* To simulate a continuous learning paradigm, model performance was monitored over increasing amounts of training data. Initially, 10% of source concepts were randomly sampled to form a test set of 227 concepts. This dataset will be referred to as the hold-out test set, and will also be used in experiment 2, the real-world evaluation. The remaining 2,034 concepts were used for training and validation. At each step an increasing fraction of data (0%–100% in steps of 20%) was randomly sampled and used as training data, with the remaining data acting as a validation set. This process was repeated 5 times per step, and the average accuracy was computed on the validation and hold-out test set. Model training was performed using a contrastive loss function. Hyperparameter tuning was performed using 20% of training data, taking number of epochs (*n_epochs_*), number of positive samples (*n_pos_*) and number of negative samples (*n_neg_*) as hyperparameters. As there is only 1 correct positive sample per source concept, data augmentation was applied by shuffling words in the laboratory test name to obtain further samples. For example, a source concept of “Blood Haemoglobin” would be shuffled and reintroduced as “Haemoglobin Blood”. If the concept only contained one word, then it would be included as an identical additional sample (upsampling). The optimal parameter set was found to be *n_epochs_*=5, *n_pos_*=1, *n_neg_*=100 based on a combined *acc_top1_* and *acc_top5_*, see Supplementary Figure 1 for full details. The larger embedding models, Qwen3 and Gemma, were trained using Low-Rank Adaptation (LoRA) (22) on a GPU machine due to the high number of parameters. Evaluation was restricted to laboratory test names, as baseline performance for medication names was already sufficiently high in scenario 1. Evaluation was only performed for the embedding models, excluding Claude 3.7.

*Scenario 3 (S3) – Onboarding a New Healthcare Provider:* To mimic onboarding of a new data source (e.g., a new hospital), data from one hospital was held out as a test set while the model was trained on data from all other hospitals. This process was repeated for each hospital, and performance metrics were averaged across iterations.

#### 2.4.2 Experiment 2 - real-world evaluation

The objective of this experiment was to evaluate the performance and usability of ArcMAP in a real-world deployment setting, from both technical and health informatician perspectives. For this purpose, ArcMAP was deployed using the fine-tuned prediction model trained and validated in Experiment 1, Scenario 2.

Over a period of 7 weeks, a clinical informatician conducted ten 30-minute time-controlled mapping sessions with the aim of validating as many laboratory test concept mappings as possible in that time. In addition to performing mappings, the informatician was asked to take structured notes after each session to provide qualitative insights into usability and identify potential challenges for future improvements. During this period, further regular mapping sessions were carried out, and additional source concepts were ingested as required by ongoing research project demands.

To investigate the efficiency gain compared with a basic mapping process, the same informatician also performed three 30 min manual mapping sessions without using ArcMAP. For these sessions, the informatician used Microsoft Excel to complete mappings. The previously mapped set of laboratory test source concepts was reused and randomly ordered. The informatician was asked to provide the correct target concept for each source concept in the adjacent column.

**Evaluation Metrics:** To assess the effectiveness of the data standardization pipeline under real-world conditions and to gain further insights about the challenges during the task, different performance metrics were recorded after each session. These metrics were designed to capture both the efficiency and challenges of the mapping process and the behaviour of the continuous learning paradigm. The metrics included:

- Concept level metrics:
  - Mapped concepts (*n_map_*): Number of concepts successfully mapped or marked as not relevant during the session
  - Skipped concepts (n_skipped_): Number of source concepts which were skipped by the user as requiring significant additional work (>10 min) for example those without a corresponding target concept or requiring further communication with the hospital
  - Not relevant concepts (*n_not_*_−rel_): Number of concepts marked as not relevant during the mapping session.
  - Concepts requiring further data analysis (n_data_): Number of concepts which were mapped in the session, but which required further data analysis (e.g., exploration of the respective laboratory values and units)
  - Difficult concepts (n_difficult_): Number of concepts that required 5–10 min of work (e.g., for searching for additional information about the source and potential target concepts). Note, each of these concepts requires normally also some kind of further data exploration and is therefore also a part of n_data_
- Prediction accuracy metrics:
  - Top-1 and Top-5 Accuracy (*acc_top1_* & *acc_top5_*): as defined in Experiment 1.
  - Cosine similarity (cs): Mean cosine similarity score between source and target concept embeddings

## 3 Results

### 3.1 Experiment 1 – model evaluation

Table 1 shows the results of experiment 1 – scenario 1 which evaluates the baseline performance of the models without further finetuning. The *acc_top1_* and *acc_top5_* obtained from each embedding model when applied to all medication and lab test names. Overall, the BioLORD model outperforms other embedding models, achieving an *acc_top1_* of 92.9% for medications and 37.0% laboratory test names, compared to 91.2% and 27.9% obtained by the next highest embedding model (SapBERT). However, the generative approach using Claude 3.7 proved to be more effective than the embeddings models when applied to laboratory tests, with an *acc_top1_*and *acc_top5_* of 43.8% and 86.7%, respectively. Accuracies weighted by concept frequency display a similar trend (Supplementary Table 1), with BioLORD achieving the highest levels compared with other embedding models (96.3% medications and 38.3% laboratory test names). However, Claude exhibits the largest accuracy gain from frequency weighting, (87.3% medications and 63.6% laboratory tests), indicating a larger proportion of its correct predictions are attributed to more frequently occurring concepts. BioLORD also outperforms other embedding models with respect to ranking quality (Supplementary Table 1), MRR=0.941 and NDCG@5 = 0.946 on medications and MRR=0.497 and NDCG@5 = 0.547 on laboratory tests. Claude again achieves a higher performance on laboratory tests (MRR=0.619 and NDCG@5 = 0.680) but not medication names (MRR=0.809 and NDCG@5 = 0.826).

**Table 1:** Baseline performance of embedding models and Claude3.7 on medications and laboratory names with 95% CI (experiment 1 – scenario 1).

Model	Medications (*n* = 6,179)		Lab tests (*n* = 2,261)	
	Top 1	Top 5	Top 1	Top5
BioLORD (ArcMAP)	**92.9% [92.7, 93.0]**	**96.1% [96.0, 96.2]**	37.0% [36.6, 37.3]	69.4% [69.0, 69.8]
SapBERT	91.2% [91.1, 91.3]	95.6% [95.5, 95.7]	27.9% [27.5, 28.2]	62.6% [62.1, 63.1]
KRISSBERT	88.5% [88.3, 88.7]	93.6 [93.4, 93.7]	20.0% [19.8, 20.2]	57.3% [56.9, 57.7]
CODER	86.1% [85.9, 86. 3]	90.7 [90.5, 90.9]	19.6% [19.3, 19.9]	48.5% [48.1, 49.0]
Gemma	71.9% [71.7, 72.0]	81.8% [81.7, 81.9]	11.7% [11.5, 11.9]	23.8% [23.4, 24.2]
Qwen3	85.6% [85.5, 85.7]	94.5% [94.4, 94.6]	23.7% [23.5, 23.9]	57.1% [56.7, 57.4]
Claude 3.7	76.6% [76.4, 76.7]	90.4% [90.4, 90.5]	**43.8% [43.5, 44.2]**	**86.7% [86.5, 87.0]**

Bold text indicates the best result per data category (medications and lab tests).

All models also perform significantly better when applied to medication names compared to laboratory tests. This disparity can be attributed to the way in which these data items are recorded in hospitals. Table 2 shows some illustrative examples of source to target concept mappings within each data category. Medication names are generally recorded using full chemical terminology or brand names, creating distinct concepts that are more easily mapped to targets. Laboratory tests on the other hand, can be highly abbreviated and encodings for each concept vary significantly across hospitals. This is further evidenced by Table 3, which shows the performance of the BioLORD model on each individual hospital. Standard deviation in *acc_top1_* and *acc_top5_* across hospitals is significantly higher for laboratory tests (7.9% and 9.9%) compared to medication names (5.4% and 3.9%). Due to the high baseline performance on medication names, the remaining analyses will focus solely on laboratory test names.

**Table 2:** Illustrative examples of medication and lab test source and target concept mappings. For each target concept, the number of source concepts that map to the concept are shown, along with 5 examples.

Target concept	Source concept count	Source concept examples
Medications		
Insulin human	102	humalog kwik pen
		humulin i
		humulin m3
		humulin s
		insulatard
Sodium chloride	45	buffered sodium chloride w/v +
		sodium chloride
		sodium chloride fpe1322
		sodium chloride freeflex
		sodium chloride viaflo
Hydrocortisone	42	hydrocortisone
		hydrocortisone acetate
		hydrocortisone acetate foam
		hydrocortisone sodium phosphate
		hydrocortisone sodium succinate
Methotrexate	29	methotrexate
		methotrexate pfp
		methotrexate syringe
		methotrexate tablets
		methotrexate intrathecal
Tacrolimus	28	tacrolimus,
		tacrolimus in wsp
		tacrolimus ointment
		tacrolimus capsules
		tacrolimus granules for oral suspension
Lab tests		
Creatinine substance concentration in serum	48	creatinine level blood
		chem 16 profile cm14 crea
		creatinine plain creup creup
		ue lft bone hitl crea
		creatinine ccr
Haemoglobin mass concentration in blood	34	fbc blocked zfbc hb
		fbc fbc hb
		full blood count hs xhcb xchc
		haemoglobin hb
		haemoglobin hgb
Red blood cell count	24	fbc rbc rbc
		full blood count hs xhcb xrbc
		rbc count rbc
		red blood cell count irbc
		red cells crc
INR - international normalised ratio	23	inr inr
		coagulation screen coag inr
		internation norm ratio inr
		prothrombin time xpt xinr
		inr linr
Platelet count in blood	23	fbc fbc plts
		full blood count hs xhcb xplt
		citrate platelet count pltc
		plt count plt
		platelets plt

**Table 3:** Performance of the BioLORD model used as prediction model in ArcMAP on medications and laboratory names for each hospital (experiment 1 – scenario 1).

Hospital	Medications		Lab tests	
	Top 1 (std=5.4)	Top 5 (std=3.9)	Top 1 (std=7.9)	Top5 (std=9.9)
Hospital 1	2,310/2,443 (94.6%)	2,377/2,443 (97.3%)	341/1,110 (30.7%)	684/1,110 (61.6%)
Hospital 2	853/922 (92.5%)	886/922 (96.1%)	3/4 (75.0%)	4/4 (100.0%)
Hospital 3	1,410/1,708 (82.6%)	1,514/1,708 (88.6%)	106/200 (53.0%)	176/200 (88.0%)
Hospital 4	1,471/1,512 (97.3%)	1,497/1,512 (99.0%)	228/493 (46.2%)	406/493 (82.4%)
Hospital 5	1,765/1,824 (96.8%)	1,807/1,824 (99.1%)	176/503 (35.0%)	334/503 (66.4%)

**Figure 4:**
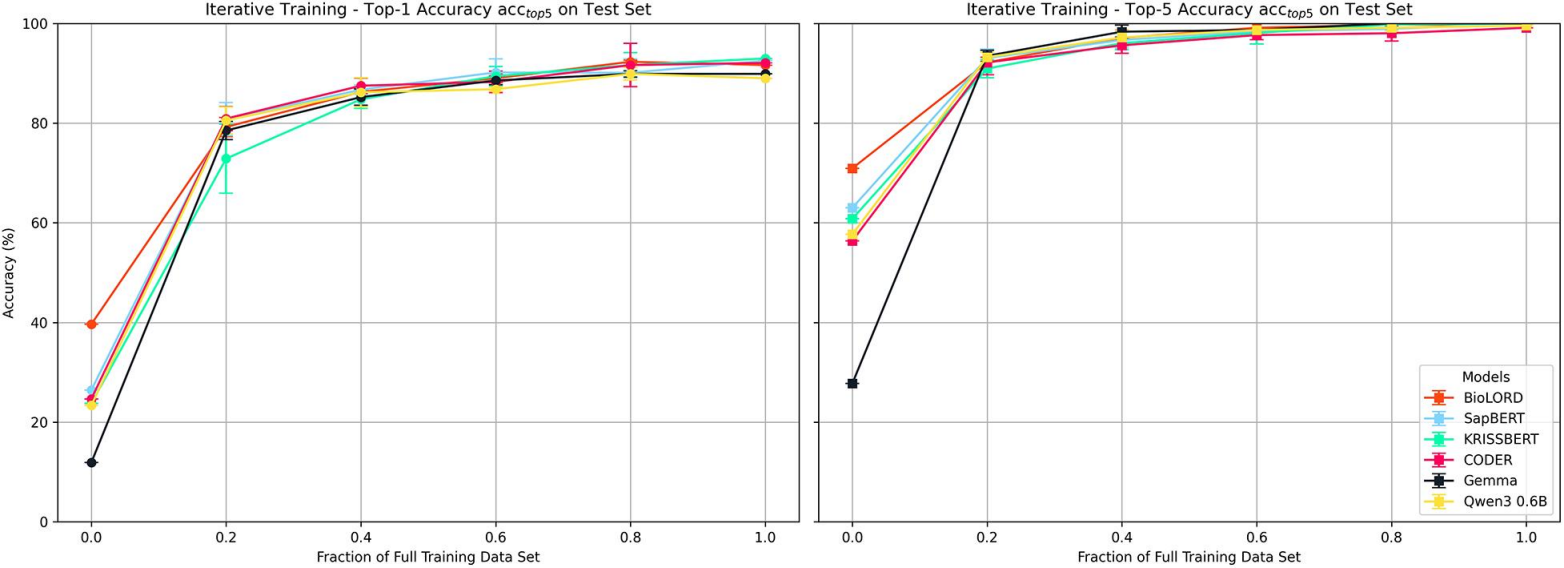
Comparison of model prediction accuracy for laboratory test names on held out test set for increasing portions of training data (experiment 1 – scenario 2). Error bars indicate std.

Figure 4 shows the average *acc_top1_* (left) and *acc_top5_* (right) for each model applied to the hold-out test set for increasing portions of training data (Exp1–S2). Results demonstrate the effectiveness of finetuning on even a small amount of data, with all models reaching between 72.9 and 80.9% *acc_top1_* and 90.9–93.5% *acc_top5_* with only 20% of training data. Model accuracies improve further to between 89.0 and 93.0% *acc_top1_* and 99.6%–100% *acc_top5_* when training on all data, highlighting the model's ability to learn hospital specific encodings. While BioLORD achieves significantly higher accuracies in the baseline case, training the other models results in substantial performance gains which brings them to the same levels of accuracy as the finetuned BioLORD model, with KRISSBERT and SapBERT even achieving marginally higher *acc_top1_* (93.0% and 92.5%) compared to BioLORD (91.6%). However, weighting the accuracies by concept frequency results in a higher *acc_top1_* for BioLORD (99.1%) compared to the next highest model, CODER (98.8%) Supplementary Figure 3. Performance on the validation sets display a similar trend, with *acc_top1_* reaching between 89.1 and 91.5% on the final validation run using 80% of training data (Supplementary Figures 4, 5). Finetuning the embeddings models also brings performance levels significantly above that of the generative approach using Claude 3.7.

**Figure 5:**
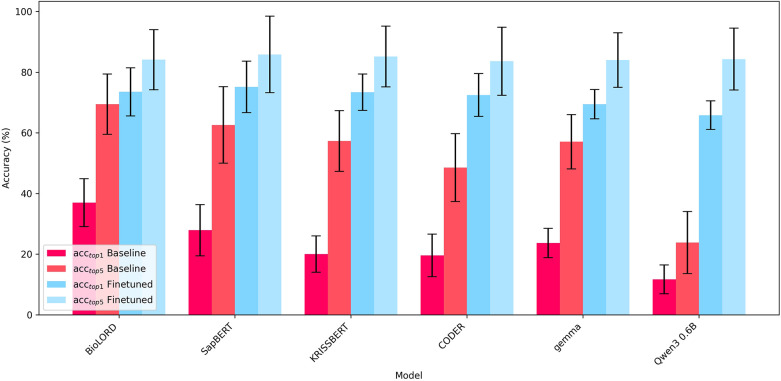
Average prediction accuracies of baseline and finetuned models for laboratory test names to predict source concepts of a new healthcare provider (experiment 1 – scenario 3). Finetuned models were trained on data of remaining healthcare providers.

Figure 5 shows the accuracy of each model to predict concepts of a new healthcare provider which wasn't part of the training set (Exp1–S3). Accuracies were computed by taking a weighted average over each hospital based on the number of concepts in that hospital (see Table 4 for exact figures). All finetuned models exhibited an improvement in performance over the baseline case, with *acc_top1_* increasing from 11.7 to 37.0% up to 65.8–75.2%. While all models improved significantly, SapBERT was the highest performing model with respect to top-1 accuracy with an *acc_top1_*of 75.2%. Evidently, finetuning the embedding models on data from other hospitals improves the model's ability to predict relevant target concepts for data from a new hospital system, despite the models never being trained on concepts from that healthcare provider. We suggest this can be attributed to similar coding regimes in certain hospitals, or the fact that some healthcare providers may use more coherent concept names to begin with. This is evidenced by Table 5, which shows the predictive accuracies of the BioLORD model on each provider before and after finetuning. The higher *acc_top1_* of hospital 3 (53.0%) and 4 (46.2%) compared to hospital 1 (30.7%) and 2 (35.5%) indicate the hospitals use concepts that are easier to map in the baseline scenario. Furthermore, hospitals 3, 4 and 5 benefit more substantially from finetuning, with an increase in *acc_top1_* of between 42% and 50%, compared to hospital 1 (increase of 27.9%). This suggests that hospital 1 uses a more distinct coding scheme for its data and the model does not benefit as greatly by training on examples from other hospitals.

**Table 4:** Performance of embedding models when evaluated on a hold-out test hospital and trained on the remaining hospitals. Accuracies are averaged over each hospital, weighted by number of samples in each hospital (experiment 1 – scenario 3).

Model	Baseline		Finetuned	
	Top 1	Top 5	Top 1	Top 5
BioLORD	**37.0% [36.6, 37.3]**	**69.4% [69.0, 69.8]**	73.5% [72.9, 74.2]	84.1% [83.6, 84.6]
SapBERT	27.9% [27.5, 28.2]	62.6% [62.1, 63.1]	**75.2% [74.6, 75.7]**	**85.8% [85.4, 86.3]**
KRISSBERT	20.0% [19.8, 20.2]	57.3% [56.9, 57.7]	73.4% [72.8, 74.0]	85.2% [84.7, 85.6]
CODER	19.6% [19.3, 19.9]	48.5% [48.1, 49.0]	72.5% [71.9, 73.1]	83.6% [83.1, 84.1]
Qwen3 0.6B	11.7% [11.5, 11.9]	23.8% [23.4, 24.2]	69.4% [68.9, 69.9]	84.0% [83.5, 84.5]
Gemma	23.7% [23.5, 23.9]	57.1% [56.7, 57.4]	65.8% [65.2, 66.5]	84.3% [83.8, 84.8]

Bold text indicates the best result per model category (baseline and finetuned).

**Table 5:** Performance of BioLORD model evaluated on a hold-out test hospital and trained on the remaining hospitals. Accuracies are shown for each individual hospital experiment 1 – scenario 3.

Hospital	Baseline		Finetuned	
	Top 1	Top 5	Top 1	Top 5
Hospital 1	341/1,110 (30.7%)	684/1,110 (61.6%)	650/1,110 (58.6%)	809/1,110 (72.9%)
Hospital 2	3/4 (75.0%)	4/4 (100.0%)	1/4 (25.0%)	1/4 (25.0%)
Hospital 3	106/200 (53.0%)	176/200 (88.0%)	184/200 (92.0%)	196/200 (98.0%)
Hospital 4	228/493 (46.2%)	406/493 (82.4%)	475/493 (96.3%)	490/493 (99.4%)
Hospital 5	176/503 (35.0%)	334/503 (66.4%)	388/503 (77.1%)	447/503 (88.9%)

Overall, BioLORD demonstrates a clear advantage over other embedding models in the baseline (non-finetuned) setting, and although Claude 3.7 achieves stronger baseline performance on laboratory tests, its weaker performance on medication names and substantially higher computational cost limit its practical utility. Furthermore, finetuning enables embedding models to surpass Claude's performance, and while SapBERT and KRISSBERT marginally outperform BioLORD in certain finetuned scenarios, these gains are small, motivating the selection of BioLORD for subsequent stages of evaluation.

### 3.2 Experiment 2 – real-world evaluation

A total of 646 concepts were mapped across the ten mapping sessions, with per-session counts ranging from 23 to 109 concepts (an average of 64.6 ± 30) (Table 6). In contrast, during the manual mapping sessions without ArcMAP, only 31.7 ± 0.9 (Supplementary Table 2) concepts were mapped on average, resulting in an estimated >2-fold increase in efficiency when using ArcMAP. These findings highlight the substantial time-saving potential of the tool compared with traditional spreadsheet-based workflows. To give a better idea of how the mapping process works in practice, Table 7 displays a list of illustrative examples to go with each concept category outlined in Section 2.4.2, accompanied with model prediction and informatician comments.

**Table 6:** Performance metrics of real-world deployment experiment.

Mapping session	Num. mappings	Concepts requiring data analysis	Num. difficult mappings	Num. skipped mappings	BioLORD Finetuned	
					Top 1	Top 5
1	23	7	1	37	90.0%	90.0%
2	32	8	2	23	56.7%	60.0%
3	88	11	0	53	80.3%	94.7%
4	51	7	2	23	85.0%	100.0%
5	93	11	1	26	93.9%	100.0%
6	109	8	0	46	86.4%	90.9%
7	62	7	0	220	91.7%	97.9%
8	88	9	0	46	95.3%	96.9%
9	70	11	0	39	98.1%	98.1%
10	30	9	1	19	84.2%	89.5%

**Table 7:** Illustrative examples of concept categories in the real-world deployment experiment.

Category	Description	Example source concept	Model prediction	Informatician decision
Not relevant concepts	Concepts that do not require mapping as they are not relevant to the category of concepts.	SAMPLE_STORED_STOR	Streptococus pneumoniae antigen test in serum quantitative result	This is not a lab test, and therefore not relevant for mapping. Marked not relevant
Skipped concepts	Concepts which may require a discussion with the hospital, decision making which is longer than the time frame available for the experiment, or which are not currently being mapped but may be mapped in the future.	RATIO_600_61	Platelet/neutrophil count ratio in blood	Without further context, it is impossible to know what type of ratio this is referring to. It is therefore necessary to skip this concept, and contact the hosptial to understand what test this source concept name represents.
Concepts requiring further data analysis	Concepts where it is not obvious from the name alone as to what it should be mapped to, and require looking at other clues in the data (e.g., range of values, unit).	U SODIUM_RAND_363_61	Sodium substance concentration in urine	While it is likely that the ‘U’ in the name indicates this is urinary sodium, it is important to check the range of values in the data aligns with this, and that this fits with the naming convention of other urine tests for that particular trust. It did and so the model suggestion was chosen.
Difficult concepts	Concepts which require further exploration of the data, taking 5–10 min in order to map correctly.	Clotting screen, blood_Prothrombin time and INR, blood	Prothrombin time	From the name, it is not clear whether this is prothrombin time, INR, or a panel rather than individual test. Data analysis was required to understand naming conventions for the trust, looking at all source concepts in clotting screens, explore the range of values for each, and look at the units. This led to strong evidence that this was in fact ‘INR - international normalised ratio’ which was chosen.

**Figure 6:**
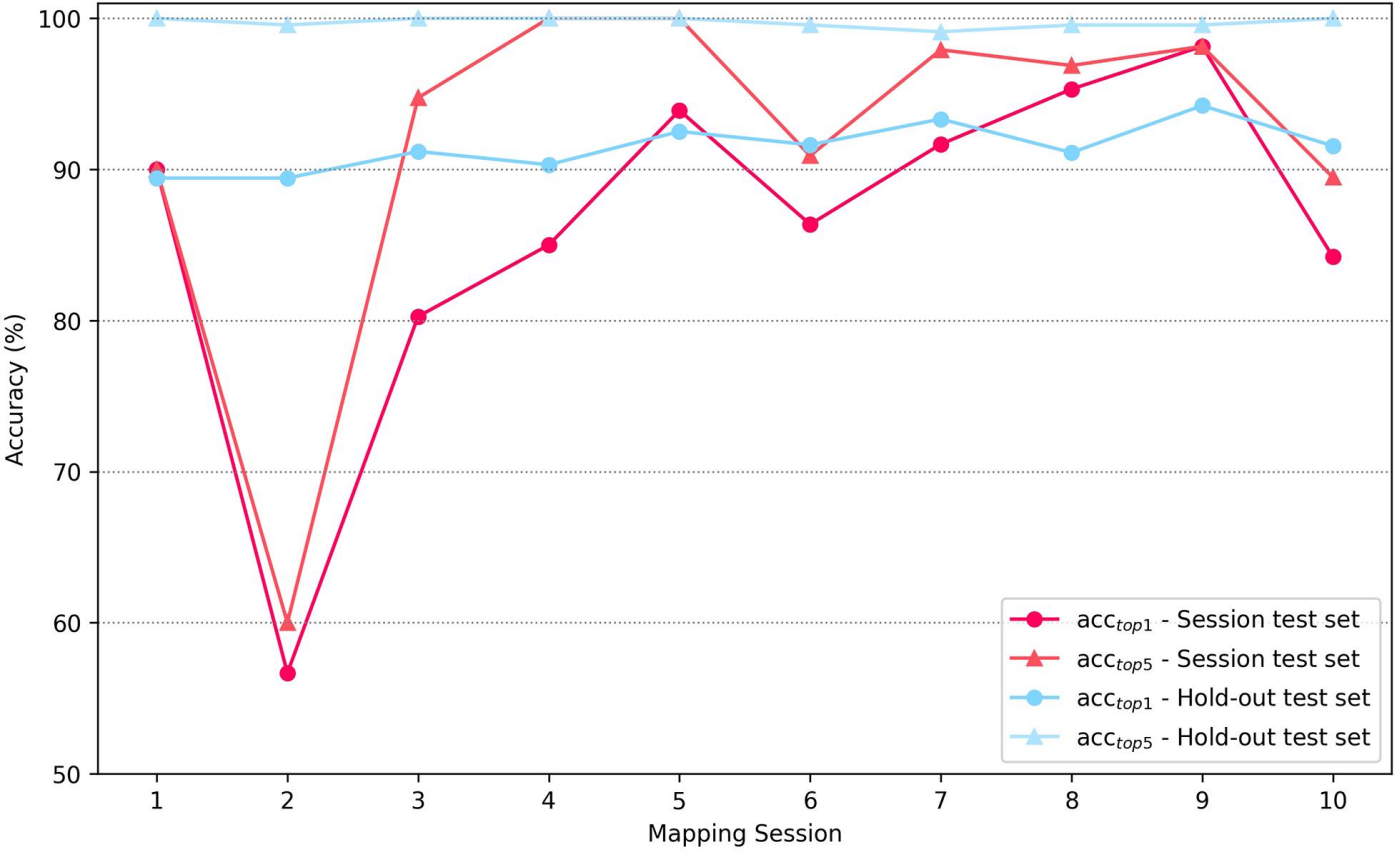
Prediction accuracies of the BioLORD model during the real-world deployment evaluation. Top-1 and top-5 accuracies (acc_top1_ and acc_top5_) presented for the static hold-out test set and for the source codes mapped in the specific session (session test set) – experiment 2.

Figure 6 illustrates the top-1 and top-5 prediction accuracy during real-world deployment, evaluated on both the general hold-out test set and the session-specific test sets. The general hold-out test set corresponds to the static test set used in Exp1-S2 and remains unchanged across all mapping sessions. In contrast, the session-specific test set comprises only the source concepts mapped during each individual session. Consequently, larger variations in accuracy are expected for the session-specific results due to differences in sample size and varying concept complexity. On the hold-out test set, top-1 accuracy displayed a gradual linear increase, starting at 89.43% in session 1 and increasing with further fine-tuning to 94.22% by session 9, reflecting the benefits of continuous learning. This was confirmed with a simple linear regression conducted over all mapping sessions (*n* = 10). The predictor **X** (mapping session) significantly predicted **Y** (top-1 accuracy), *β*=0.367, SE = 0.129, t(8) = 2.85, *p* = 0.021, explaining 50% of the variance (R^2^ = 0.50). In contrast, top-1 accuracy on session-specific test sets exhibited greater variability, ranging from 80.26% to 98.15%, with one notable outlier in session 2 (*acc_top1_* = 56.7%). Top-5 accuracy on the hold-out test set was stable, consistently between 99% and 100%, which is in line with results of Exp1-S2. For session-specific test sets, top-5 accuracy was generally robust, remaining above 90% for all sessions except session 2 and frequently reaching 100%, indicating that the correct concept was almost always among the top five suggestions.

Table 8 shows the average cosine similarities between the source concept and the closest target concept for correct (0.97 ± 0.06) and incorrect (0.81 ± 0.14) predictions. Results indicate a significant difference in mean similarity score under Welch's *t*-test (t-statistic: 8.8 *p*-value <0.01), highlighting the model's ability to quantify uncertainty in its predictions.

**Table 8:** Average cosine similarity scores between correct and incorrect predictions.

Prediction	Num samples	Mean similarity	Std similarity
Incorrect	64	0.81	0.14
Correct	457	0.97	0.06

**Figure 7:**
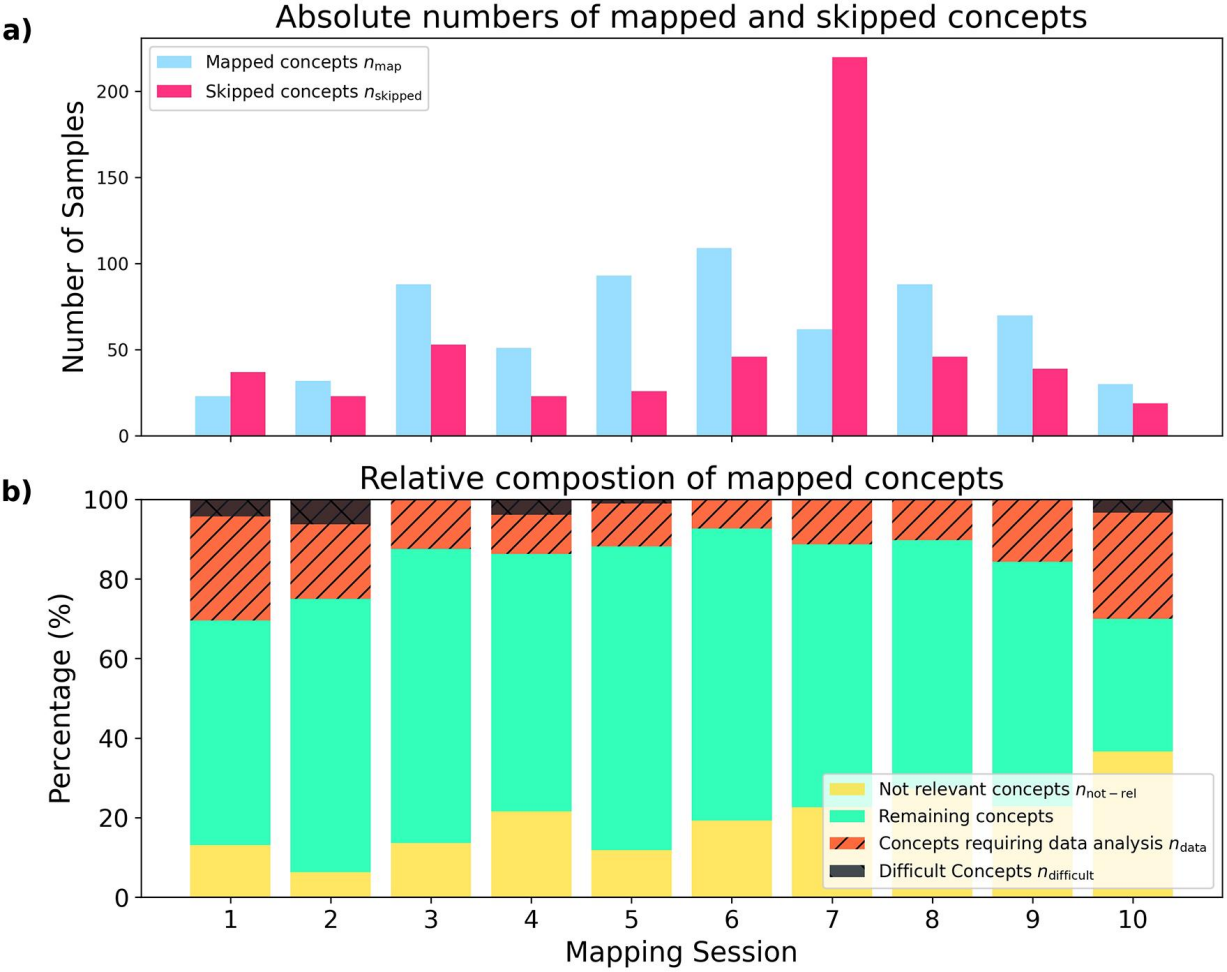
**(a)** Absolute number of mapped and skipped concepts for each 30 min mapping session; **(b)** relative percentage of concepts marked as not relevant, difficult (requiring 5−10mins of investigations) or concepts which required further data analysis relative to the amount of mapped concepts per session.

Figure 7 provides a detailed overview of the concept-level metrics observed during the ten 30-minute mapping sessions using ArcMAP. Figure 7a shows the absolute number of source concepts mapped per session alongside the number of concepts skipped. A substantial variation is evident across sessions, with mapped concepts ranging widely and skipped concepts reaching up to 220 in one session, indicating differences in data complexity and workload.

Figure 7b illustrates the relative composition of mapped concepts, categorised as *not relevant*, *difficult* (requiring 5–10 min of additional investigation), and those requiring further data analysis (e.g., review of laboratory values and units). Again, considerable variability is observed between sessions, and no clear trend emerges over time. On average, 16.8% of mapped concepts required further data analysis and 19.5% were marked as *not relevant*. The proportion of concepts marked as *not relevant* appeared to increase over time, rising from 6.25% in session 2 to 36.7% in session 10, suggesting evolving data characteristics or changes in mapping priorities. In approximately half of the sessions, at least one difficult concept was encountered, requiring 5–10 min of additional work—a substantial effort within a 30-minute session.

These findings highlight the heterogeneity of mapping tasks in real-world settings and underscore the importance of adaptive workflows to handle varying complexity.

## 4 Discussion

ArcMAP was developed to facilitate data standardisation by combining machine learning with an intuitive graphical user interface. The application focuses on the data standardisation with data coming from UK based hospital systems. Beyond increased mapping efficiency, the tool reduces the risk of copy-paste errors, mitigates version control issues, and streamlines the search process by presenting the most likely target concepts directly to the user.

Despite the strong performance of biomedical embedding models reported in prior work, the low baseline accuracy observed for laboratory test standardisation in Experiment 1, Scenario 1 was initially surprising. Models such as SapBERT, CODER, KRISSBERT, and BioLORD have consistently demonstrated high top-1 accuracies in their respective publications, often exceeding 80%–90% on standard biomedical concept normalisation and entity linking benchmarks. However, a closer inspection of the evaluation settings used in these studies indicates an important domain mismatch that helps explain the discrepancy observed in our results.

Across prior studies, model performance is predominantly evaluated on datasets derived from biomedical literature or curated clinical text, including NCBI Disease, BC5CDR, MedMentions, CADEC, PsyTAR, and related benchmarks. These datasets are largely based on PubMed abstracts, scientific articles, discharge summaries, or patient-generated text, and focus primarily on disease, chemical, drug, or adverse event entities. In contrast, the laboratory test names available in this study exhibit substantially different characteristics.

As illustrated in, a single laboratory concept may appear under numerous abbreviated or institution-specific surface forms that share little lexical overlap with their standardized target concepts. These names are frequently short, highly abbreviated, locally defined, and sparsely contextualised rather than descriptive phrases. Consequently, baseline top-1 accuracies remain below 40%, underscoring that pre-training on biomedical literature and ontology definitions alone is insufficient to capture the extreme variability and abbreviation patterns encountered in laboratory test naming.

Taken together, these findings highlight a gap between commonly used benchmark datasets and real-world data standardisation tasks. Under such conditions, even models specifically designed for biomedical concept representation struggle to generalise without exposure to domain- and institution-specific examples.

Experiment 1 – session 2 demonstrates the impact of fine-tuning on the accuracy of clinical concept standardisation. Across our experiments, fine-tuning consistently improved baseline performance, with all evaluated domain-embedding models—SapBERT, KRISSBERT, CODER, and BioLORD—achieving top-1 accuracies above 91% when trained on the full domain-specific dataset, and top-5 accuracies approaching or reaching 100% (Figure 4).

While substantial performance differences are observed in the non-fine-tuned setting, these differences rapidly diminish as training data is introduced. Even with moderate amounts of domain-specific supervision, model performance converges, and the variance between architectures becomes small. When fine-tuned on the complete dataset, all four investigated biomedical embedding models achieve comparable results, indicating that adaptation to the target domain is far more influential than the selection of a particular pre-trained model.

In the baseline comparison, the LLM Claude 3.7 slightly outperformed the smaller embedding models (Table 1). However, once finetuned, the smaller embedding models demonstrated superior performance. For example, top-1 accuracy was 37.0%, 43.8%, and 91.6% for non-finetuned BioLORD, Claude 3.7, and fine-tuned BioLORD respectively. These results align with findings by (23) who reported that smaller models, when finetuned, are superior for tasks that do not require text generation, such as concept mapping. Although prompt engineering strategies (e.g., few-shot or chain-of-thought prompting) could further improve LLM performance, the size difference between Claude 3.7 (>100 billion parameters) and BioLORD (<1 billion) and the already good performance of small embedding models makes the use of LLMs unjustifiable for this task given their high hardware requirements and associated costs.

Evaluating the onboarding of a new hospital (Exp1-S3) revealed a reduction in mean performance, with BioLORD's top-1 accuracy decreasing from 91.6% in the fully fine-tuned setting to 73.5%. Similar trends were observed for SapBERT, CODER, and KRISSBERT. This decline is primarily driven by substantial variability in laboratory test naming conventions across hospitals, as illustrated by the example source concepts in Table 2.

Importantly, the mean top-1 accuracy of 73.5% remains substantially higher than the baseline performance of 37.0%, indicating that fine-tuning on hospital data from a similar geographical region already provides meaningful adaptation. Nevertheless, consistent with the learning behaviour observed in Experiment 1, Scenario 2, we expect that even a small amount of hospital-specific training data would further and significantly improve mapping accuracy during onboarding.

The real-world evaluation aimed to assess ArcMAP's performance in a production setting. Over seven weeks, ten mapping sessions were conducted alongside normal project specific day to day work. This meant that other research project specific mappings were performed and new data ingested between mapping sessions. From a machine learning perspective, prediction accuracy continued to improve despite the relatively small amount of new training data (646 concepts compared with >2,500 in the initial fine-tuning dataset). This increase was captured by a linear regression model on the top-1 accuracy on the hold-out test set.

The efficiency measured by the number of mapped concepts as well as accuracy of the prediction algorithm for session-specific test sets, varied considerably across mapping sessions. This variability can be attributed to several factors:

- **Interpretability of source concept names:** A major factor affecting the number of concepts which could be mapped in a single session was the interpretability of the source names. For example, a source concept name of ‘Total protein level, blood’ can quite easily be mapped to the target ‘Protein mass concentration in serum’. However, concepts such as ‘INV_INV_PROT’ are more difficult to interpret, and while it can be guessed that this also maps to ‘Protein mass concentration in serum’, extra time is required to check the units, value range, and naming conventions of that hospital to be sure of the mapping.
- **Sorting bias**: Source concepts were sorted by frequency for all mapping sessions, meaning that later sessions contained less common and potentially more challenging concepts. Although this ordering may appear unconventional, the large number of unique source concepts in these datasets makes frequency-based standardisation a practical strategy, as it ensures that a substantial absolute number of concepts is mapped for each project.
- **Rare concepts**: In a similar direction, it must be mentioned that the original training data was based on previous research projects and likely included more common concepts. This could impact also the prediction accuracy of the tool, tasked to deal with more challenging concepts compared to the training data.
- **Panel tests**: A panel is a group of tests performed together (e.g., full blood count or liver function test), and when these appear in the source data, mapping can be accelerated because identifying the panel type clarifies the individual tests. This explains the high number of mapped concepts in sessions 5 and 6, which contained several panels. Conversely, session 2 showed particularly low performance for both top-1 and top-5 accuracy. On closer inspection, this session included a set of panel tests with highly granular individual test names that lacked appropriate matches in the target ontology. These were all mapped to a single higher-level concept, which significantly reduced accuracy. Because source concepts were sorted by frequency, such panels appear grouped together, meaning a large panel can disproportionately influence performance in a single session.
- **Real-world impact**: Between session 6 and 7, new source data was ingested which affected the order of concepts, with new unmapped concepts scattered between previously skipped concepts. Rather than continuing mapping from the previous session, it meant that it was necessary to start again from the most frequently unmapped concept. This resulted in a large number of concepts being skipped, the majority of which were likely concepts which had been skipped in previous mapping sessions.

Compared to classical manual approaches such as spreadsheet-based mapping, ArcMAP was associated with higher mapping efficiency, with an observed increase of at least twofold. However, this comparison should be interpreted cautiously. The source concepts mapped during the manual sessions were drawn from the initial training dataset (see Section 2.3) and therefore likely represented more frequently used and more easily interpretable laboratory tests. This is reflected in the manual mapping sessions, where only 1.7 concepts on average required further data analysis (compared to 16.8 in the ArcMAP sessions), and no difficult concepts were encountered. As a result, the reported efficiency gains should be viewed as indicative rather than a definitive quantitative comparison. Nevertheless, these findings suggest that ArcMAP can meaningfully support and accelerate concept mapping in realistic deployment settings, particularly when dealing with more complex, less frequent, or institution-specific concepts.

Our results highlight the inherent challenges of standardising medical concepts and the substantial variability present in real-world data. To better understand the practical use of such a tool, we deliberately chose not to control for data heterogeneity or factors influencing session performance. While this design provides a realistic assessment of deployment conditions, it also introduces limitations. For example, user feedback indicated that the “difficult” category (5–10 min) was too coarse, and that more granular tracking of task complexity would be beneficial in future evaluations. In addition, although qualitative challenges such as abbreviations and panel tests were observed, the study design did not capture structured error categories or confidence-error relationships, limiting retrospective failure-mode analysis; addressing this will be important in future work. Finally, the inclusion of multiple annotators would enable assessment of inter-rater reliability, which was not possible in the current study.

Nevertheless, these challenges are typical of real-world data environments. Importantly, the value of ArcMAP lies in its ability to support work under these conditions. Subjective feedback from informaticians confirmed that the tool significantly improved the standardisation process compared with traditional methods, both in terms of efficiency and user experience.

Another important observation from the real-world deployment is that each mapping session contained a substantial number of concepts that either required further data analysis or were not relevant to the specific project needs. This highlights an opportunity for optimisation in future iterations of ArcMAP. One potential improvement is the incorporation of additional contextual information, such as laboratory value summary metrics (e.g., mean, minimum, maximum), to enhance accuracy for concepts that currently demand manual investigation. Extending this approach could also support the mapping of more challenging concepts by including broader clinical context, for example, diagnoses and procedures recorded during the same admission. Furthermore, training models to automatically predict concept relevance would help reduce the time spent on non-essential mappings and streamline the overall workflow. Our findings in experiment 2 show that cosine similarity can serve a reliable indicator of model confidence, as evidenced by the marked difference in similarity scores between correct and incorrect predictions. Going forward, these confidence scores could be surfaced to human reviewers to prioritize high-certainty suggestions, enabling faster validation while maintaining expert oversight.

In general, deploying a continuously updated mapping model such as ArcMAP in a production setting requires appropriate governance and safeguards. ArcMAP maintains full transparency by tracking each mapping action, including when a mapping was created or modified and by whom, thereby enabling comprehensive audit trails and retrospective review. As prediction models are updated over time and new hospitals are onboarded, monitoring for distributional shifts becomes increasingly important and will be investigated in future evaluations.

Another risk inherent to human-in-the-loop learning is the potential propagation of errors, whereby an incorrect user confirmation is incorporated into subsequent model updates. This risk is currently mitigated by restricting edit permissions to a limited group of trained users, while providing ArcMAP in a read-only mode to downstream data consumers, enabling them to identify and report potential mapping errors without directly modifying the mappings.

In summary, ArcMAP is designed to address the challenges of data standardisation in clinical environments by integrating machine learning with an intuitive graphical user interface. The evaluation presented here is based on data from five UK-based hospital systems and focuses on mapping to two standard clinical vocabularies. Our results demonstrate that fine-tuning dedicated concept-mapping models on local data is essential to achieve high accuracy, particularly for laboratory test standardisation. The real-world evaluation conducted over a two-month period provided practical insights into challenging cases that require additional contextual information, such as laboratory values or local coding conventions. Nevertheless, ArcMAP substantially increased mapping efficiency through its continuous learning pipeline and interactive interface, offering a scalable and robust solution for standardising diverse clinical concepts in real-world healthcare settings.

## Data Availability

The data analyzed in this study is subject to the following licenses/restrictions: The data used for the purposes of this paper are provided as part of a contract with our NHS partners. As such the data is not available for public access or for sharing out with the terms of our contractual agreements with our partners. Requests to access these datasets should be directed to joseph.cronin@arcturisdata.com.

## Supplementary material

Supplemental Figure 1 Results of hyperparameter tuning the BioLORD model on a 20% sample of training data across number of epochs, number of positive samples and number of negative samples.

Supplemental Figure 2 Prompt provided to the Claude 3.7 sonnet model. Model was provided with a given source concept and list of all target concepts.

Supplemental Figure 3 Comparison of model prediction accuracy weighted by concept frequency for laboratory test names on held out test set for increasing portions of training data (experiment 1 – scenario 2). Error bars indicate std.

Supplemental Figure 4 Comparison of model prediction accuracy weighted by concept frequency for laboratory test names on cross validation sets for increasing portions of training data (experiment 1 – scenario 2). Note that for training size=0.0, the baseline model performances are shown on all data. Error bars indicate std.

Supplemental Figure 5 Comparison of model prediction accuracy weighted by concept frequency for laboratory test names on cross validation sets for increasing portions of training data (experiment 1 – scenario 2). Note that for training size=0.0, the baseline model performances are shown on all data. Error bars indicate std.
