# Supplementary figures and images for: ArcMAP – ML assisted medical concept mapping to accelerate NHS data standardization
Source: Front Digit Health. 2026 Apr 14;8:1770903. doi: 10.3389/fdgth.2026.1770903 (PMC13121272; doi:10.3389/fdgth.2026.1770903)

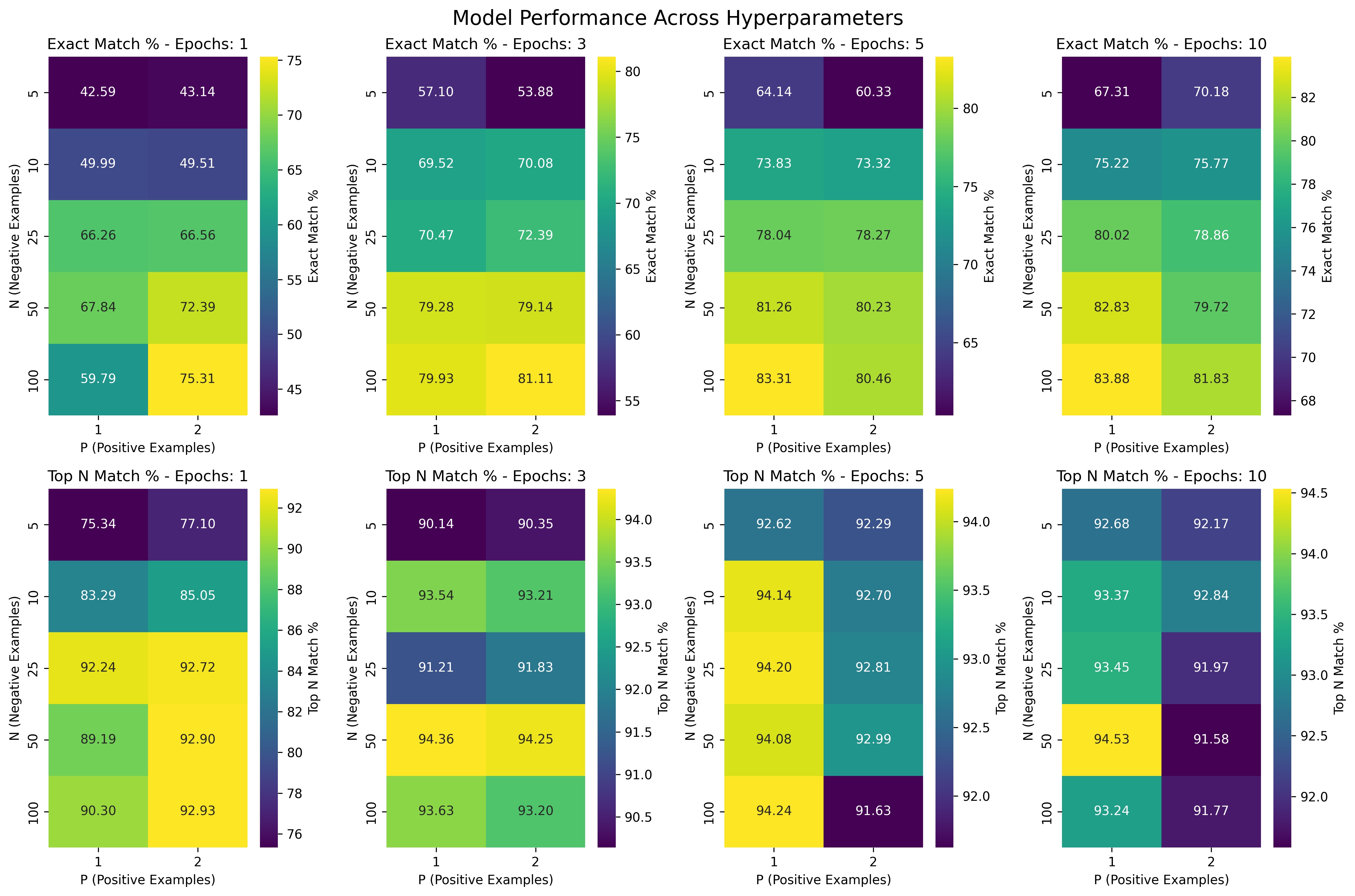

Supplement: Supplemental Figure 1 — Results of hyperparameter tuning the BioLORD model on a 20% sample of training data across number of epochs, number of positive samples and number of negative samples. [file Image1.jpeg]

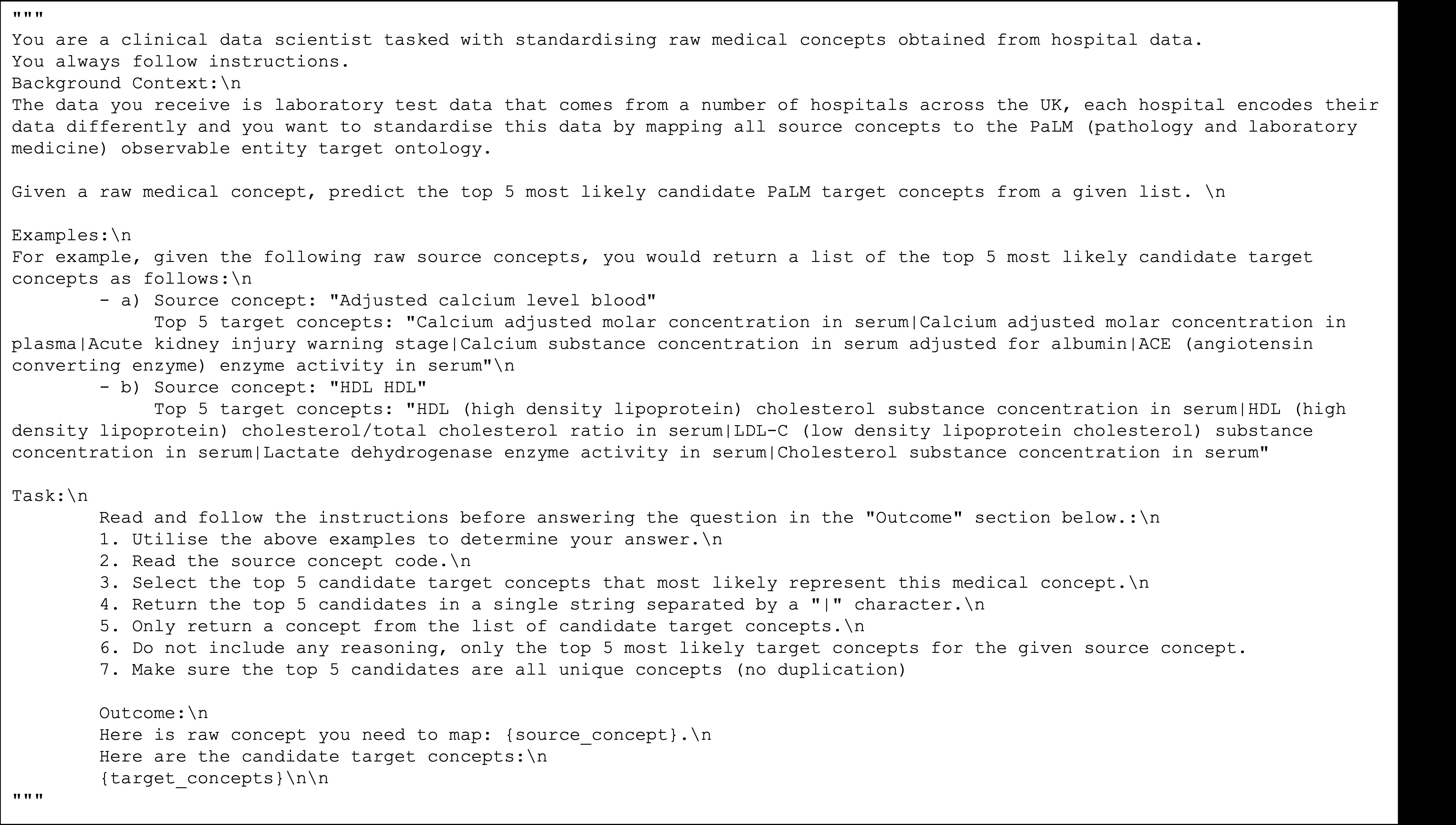

Supplement: Supplemental Figure 2 — Prompt provided to the Claude 3.7 sonnet model. Model was provided with a given source concept and list of all target concepts. [file Image2.jpeg]

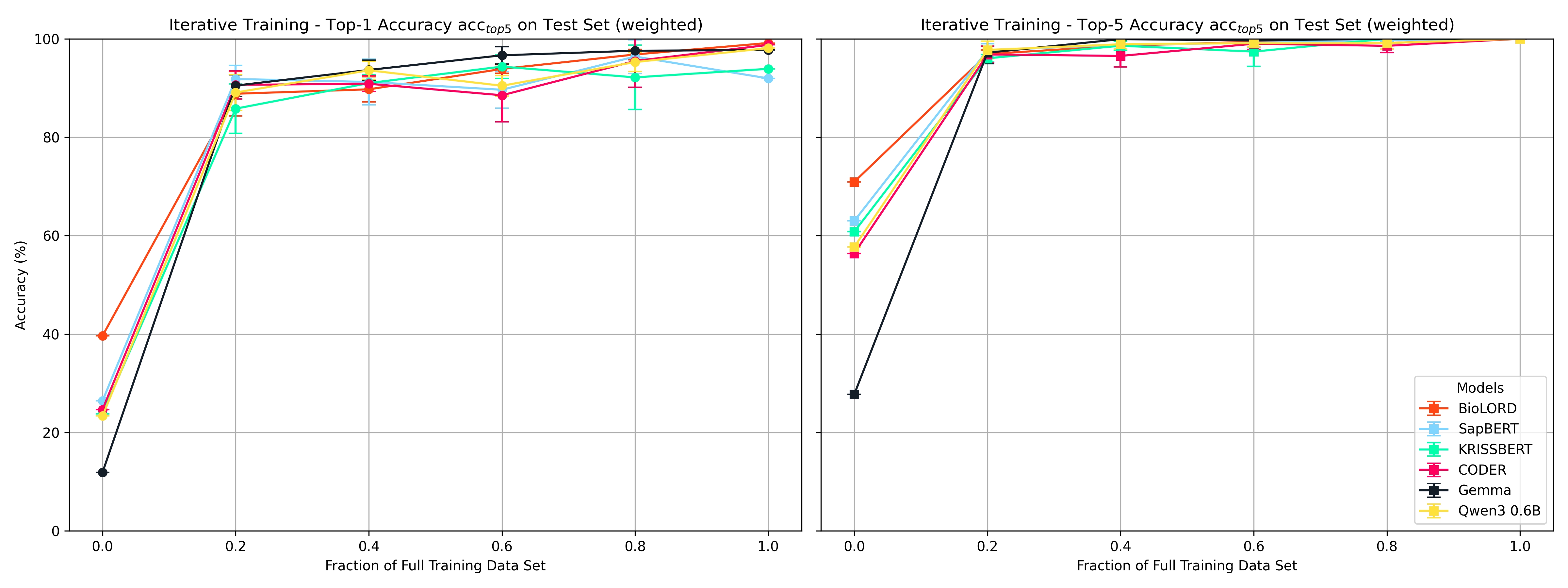

Supplement: Supplemental Figure 3 — Comparison of model prediction accuracy weighted by concept frequency for laboratory test names on held out test set for increasing portions of training data (experiment 1 – scenario 2). Error bars indicate std. [file Image3.jpeg]

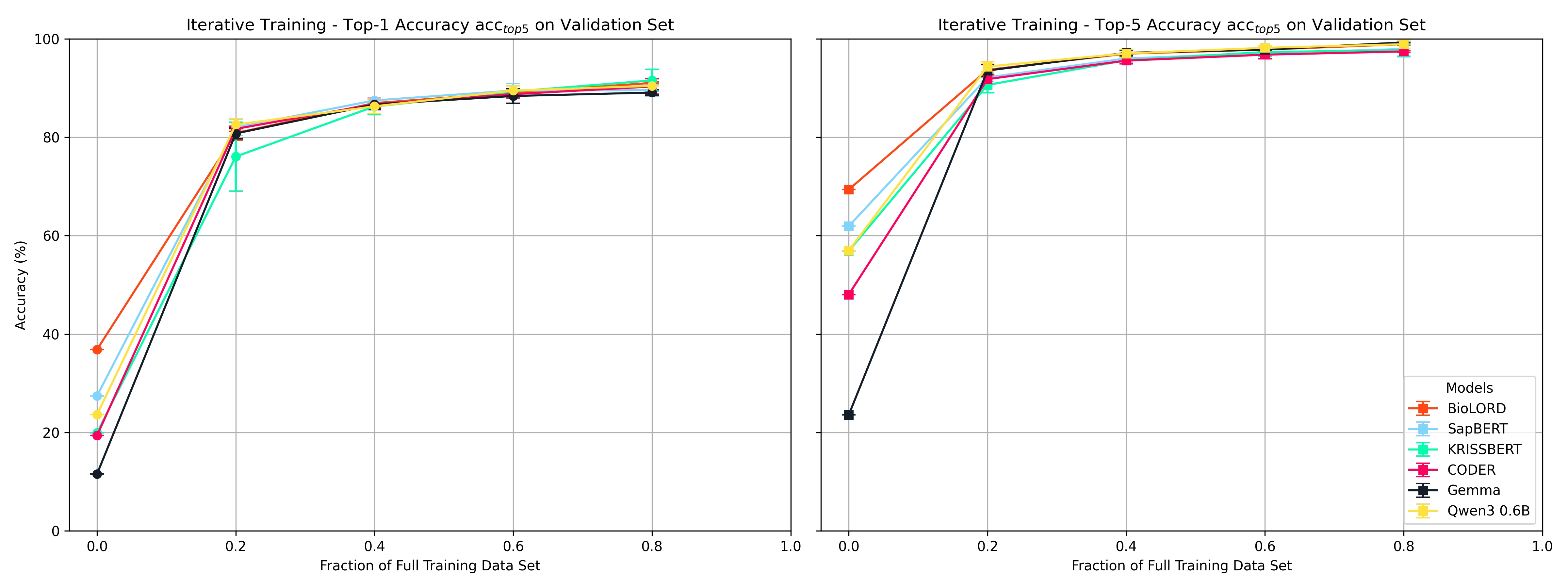

Supplement: Supplemental Figure 4 — Comparison of model prediction accuracy weighted by concept frequency for laboratory test names on cross validation sets for increasing portions of training data (experiment 1 – scenario 2). Note that for training size=0.0, the baseline model performances are shown on all data. Error bars indicate std. [file Image4.jpeg]

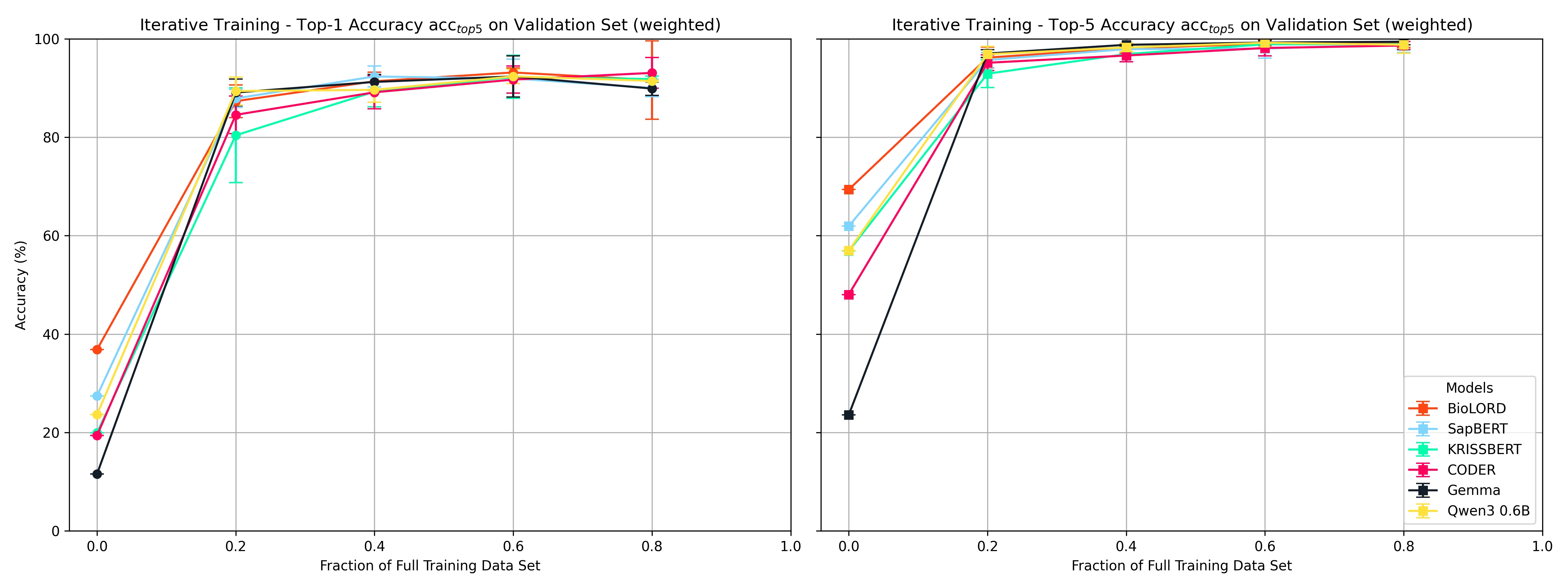

Supplement: Supplemental Figure 5 — Comparison of model prediction accuracy weighted by concept frequency for laboratory test names on cross validation sets for increasing portions of training data (experiment 1 – scenario 2). Note that for training size=0.0, the baseline model performances are shown on all data. Error bars indicate std. [file Image5.jpeg]
