# Supplementary material for: ArcMAP – ML assisted medical concept mapping to accelerate NHS data standardization
Source: Front Digit Health. 2026 Apr 14;8:1770903. doi: 10.3389/fdgth.2026.1770903 (PMC13121272; doi:10.3389/fdgth.2026.1770903)
Supplement: Supplementary file 6 [file Datasheet1.pdf]

Supplemental Table 1 – Extended baseline performance metrics of embedding models and Claude3.7 on medications and laboratory names with 95% CI (experiment 1 – scenario 1). Bold text indicates the best result per data category (medications and lab tests)

	Lab tests (n=2261)			
**Model**	**Top 1 (freq. weighted)**	**Top5 (freq. weighted)**	**MRR**	**NDCG@5**
**BioLORD (ArcMAP)**	38.3% [37.6, 39.0]	67.4% [66.9, 68.0]	0.497 [0.494, 0.501]	0.547 [0.543, 0.550]
**SapBERT**	31.8% [31.2, 32.4]	62.1% [61.3, 62.8]	0.409 [0.404, 0.413]	0.463 [0.458, 0.467]
**KRISSBERT**	17.8% [17.3, 18.4]	47.0% [46.3, 47.8]	0.336 [0.333, 0.339]	0.395 [0.391, 0.398]
**CODER**	15.9% [15.4, 16.4]	36.4% [35.5, 37.3]	0.302 [0.298, 0.305]	0.347 [0.343, 0.350]
**Gemma**	11.7% [11.5, 11.9]	23.8% [23.4, 24.2]	0.162 [0.159, 0.165]	0.180 [0.178, 0.184]
**Qwen3**	18.9% [18.6, 19.2]	55.1% [54.7, 55.4]	0.358 [0.355, 0.360]	0.411 [0.408, 0.414]
**Claude 3.7**	**63.6% [63.5, 63.8]**	**90.9% [90.8, 91.0]**	**0.619 [0.616, 0.621]**	**0.68 [0.678, 0.683]**
	**Medication (n=6179)**			
**BioLORD (ArcMAP)**	**96.3% [96.2, 96.4]**	97.4% [97.3, 97.4]	**0.941 [0.940, 0.942]**	**0.946 [0.945, 0.947]**
**SapBERT**	95.2% [95.2, 95.3]	**97.6% [97.6, 97.7]**	0.929 [0.927, 0.930]	0.934 [0.933, 0.936]
**KRISSBERT**	94.5% [94.4, 95.6]	95.5 [95.4, 95.5]	0.905 [0.904, 0.907]	0.913 [0.911, 0.914]
**CODER**	94.2% [94.0, 94.3]	95.1 [95.0, 95.2]	0.879 [0.877, 0.881	0.886 [0.884, 0.888]
**Gemma**	80.4% [80.4, 80.5]	86.0% [85.9, 86.1]	0.756 [0.754, 0.757]	0.770 [0.768, 0.771]
**Qwen3**	89.8% [89.7, 89.9]	95.4% [95.3, 95.5]	0.893 [0.893, 0.894]	0.906 [0.905, 0.907]
**Claude 3.7**	87.3% [87.3, 87.4]	96.6% [96.5, 96.6]	0.809 [0.808, 0.810]	0.826 [0.825, 0.826]

Supplemental Table 2 - Manual mapping metrics

**Mapping session**	**Num. mappings**	**Concepts requiring data analysis**	**Num. difficult mappings**	**Num. skipped mappings**
**1**	31	0	0	1
**2**	31	3	0	1
**3**	33	2	0	0
